# Inequalities in adherence to the continuum of maternal and child health service utilization in Ethiopia: multilevel analysis

**DOI:** 10.1186/s41043-021-00271-w

**Published:** 2021-10-30

**Authors:** Nigatu Regassa Geda, Cindy Xin Feng, Carol J. Henry, Rein Lepnurm, Bonnie Janzen, Susan J. Whiting

**Affiliations:** 1 Center for Population Studies, College of Development Studies, Addis Ababa University, Sidist Kilo Campus, PO Box 1176, Addis Ababa, Ethiopia; 2 School of Public Health, Health Science E-Wing, University of Saskatchewan, 104 Clinic Place, Saskatoon, SK S7N 2Z4 Canada; 3 College of Pharmacy and Nutrition, Health Sciences A-Wing, University of Saskatchewan, 107 Wiggins Road, Saskatoon, SK S7N 5E5 Canada; 4 Department of Community Health and Epidemiology, Faculty of Medicine, Dalhousie University, Halifax, Canada; 5 Department of Community Health and Epidemiology, Collège of Medicine, University of Saskatchewan, Saskatoon, Canada

**Keywords:** Antenatal care, Delivery care, Postnatal care, Service utilization, Micronutrient supplementation, Ethiopia

## Abstract

**Background:**

Despite progress made to improve access to child health services, mothers’ consistent utilization of these services has been constrained by several factors. This study is aimed at assessing the inequalities in key child health service utilization and assess the role of antenatal care (ANC) on subsequent service use.

**Method:**

The analysis of the present study was based on the Ethiopian Demographic and Health Surveys, a nationally representative sample of 10,641 children. A health service utilization score was constructed from the affirmative responses of six key child health interventions associated with the most recent birth: ANC service, delivery of the last child at health facilities, postnatal care services, vitamin A intake, iron supplementation and intake of deworming pills by the index child. A mixed effect Poisson regression model was used to examine the predictors of health service utilization and three separate mixed effect logistic regression models for assessing the role of ANC for continued use of delivery and postnatal care services.

**Results:**

The results of mixed effect Poisson regression indicate that the expected mean score of health service utilization was lower among non-first birth order children, older and high parity women, those living in polygamous families and women living in households with no access to radio. The score was higher for respondents with better education, women who had previous experience of terminated pregnancy, residing in more affluent households, and women with experiences of mild to high intimate partner violence. Further analysis of the three key health services (ANC, delivery, and postnatal care), using three models of mixed effect logistic regression, indicates consistent positive impacts of ANC on the continuum of utilizing delivery and postnatal care services. ANC had the strongest effects on both institutional delivery and postnatal care service utilization.

**Conclusion:**

The findings implicated that maternal and child health services appear as continuum actions/behavior where utilization of one affects the likelihood of the next service types. The study indicated that promoting proper ANC services is very beneficial in increasing the likelihood of mothers utilizing subsequent services such as delivery and postnatal care services.

## Background

Health services such as skilled attendance during pregnancy, institutional delivery, early postnatal checkup, and micronutrient supplementations are the most appropriate interventions in ensuring child health and survival and for attaining the Sustainable Development Goals/SDGs [1].

Antenatal care (ANC), when performed from the early stages of pregnancy, promotes regular check-ups for the health of the pregnant woman and the baby [2]. The World Health Organization/WHO [2, 3] recommends that a good quality of care is four visits for normal pregnancies and should include education, counseling, screening and treatment to monitor and promote the well-being of the mother and the fetus. Despite significant increases in ANC, the proportion of deliveries attended by skilled health personnel is still low in Sub-Saharan Africa and Southern Asia, the regions experiencing the highest numbers of maternal and child deaths [4]. Recent studies in some developing countries reported that women’s uptake of ANC by a health professional reduces dropout from maternal and child health care services [2, 5, 6]. Women who have adequate ANC visits during their pregnancy are more likely to visit health facilities for child health services such as vaccinations and nutritional supplements (iron pills and vitamin A) [7, 8]. Studies in the developing world have documented that proper utilization of the pre- and post-delivery services significantly decreases infant mortality [9–12]. However, consistently deciding to seek care is usually constrained by a wide range of sociocultural and demographic factors [13].

In Ethiopia, the prevalence of all the key components of health service utilization has been very low for every standard. For example, the last four Ethiopian Demographic and Health Surveys/EDHS indicated a gradual improvement in the proportion of mothers visiting ANC at least once before delivery, from 27% in 2000 to 63% in 2016 [14, 15]. However, only a quarter of births in Ethiopia were delivered with the assistance of skilled birth attendants[15]. In Ethiopia, antenatal and delivery care utilization are not only beneficial in terms of avoiding adverse outcomes for pregnancy or complications, but they are also important entry point for delivery of the Essential Nutrition Actions (ENA) message [16]. Other preventive health services, such as supplementation of vitamin A, iron, deworming, and immunization to children, also remained very low. For example, less than 10% of Ethiopian children aged 6–59 months received an iron supplement and deworming pills [15].

Previous studies in Ethiopia identified a range of variables affecting decisions to use the aforementioned child health services [10–12, 17]. These studies reported that residing in rural areas, having no education, being in lower wealth groups, being older, and having a higher parity were important predictors of ANC, delivery, and/or postnatal care services. However, most of these studies based their findings on a small area or a small sample, and they dealt with only a few components of child health services. In practice, any one of these services is a continuum of actions where attendance of one will affect the likelihood of adhering to the next service types. To the best of our knowledge, no study has attempted to use a more comprehensive measure of health service utilization constructed based on a complete continuum of child health care through the pre- and postnatal periods.

The current study, thus, primarily aimed to assess the socioeconomic disparities in the use of children’s health services in Ethiopia based on a composite outcome measure. For economically poor countries like Ethiopia, where women have low literacy and little access to services, it is very important to understand how receipt of ANC relates to subsequent vital health services along the continuum of care. Thus, a secondary-level analysis will address the association between ANC and uptake of the next two key health service utilizations (delivery and postnatal care) among Ethiopian women.

## Methods

### The study context

Ethiopia is the second-most populous nation in Africa with an estimated population of 109 million people [18]. Children (0–14 years) account for about 40% of the total population of the country [19]. Administratively, the country is divided into nine regions and two autonomous cities. The country has an agrarian economy, where agriculture accounts for more than 60% of the GDP and employs nearly 85% of the population[16]. According to World Bank estimates, Ethiopian economy was the third-fastest growing among those having 10 million or more population in the world (for the period 2000 to 2018), as measured by GDP per capita [20]. However, nearly a third of its population still lives below the poverty line and two-thirds have no education and limited access to health care services [21]. Despite remarkable improvements in child survival rates, both infant and child mortality rates are one of the highest in Sub-Saharan African countries [15].

Ethiopian national health policy emphasizes health care decentralization and prioritization of health promotion, disease prevention and basic curative services[22]. At the micro-level, the Essential Health Service Package (EHSP) has been used to guide service provision with a clear stratification of service delivery and financial arrangements [22]. The Ethiopian health system is a four-tier health care system, which is organized into Primary Health Care Units (PHCUs), District Hospitals, General Hospitals and Specialized Hospitals [23, 24]. Under each PHCU, there are five satellite Health Posts, each post serving approximately 5000 people. The PHC provides essential health care usually free for people living in rural areas [23, 24]. Health Extension Workers (HEW), deployed to each health post, are mandated to provide antenatal care, administer vaccines, conduct normal and safe deliveries, conduct monitoring of growth, provide nutrition counseling, offer family planning services, and organize referrals for services, hygiene and environmental sanitation, and health education and Communication [23, 24].

### Data sources

The EDHS of 2016 collected health-related information from women of reproductive ages 15–49 [15]. It is a cross-sectional household survey which employed a stratified two-stage cluster sample design. For the present analysis, the recoded data file of the EDHS, which contains entries for 10,641 respondents who had children under five years of age, was used. The EDHS data were collected from 645 enumeration areas (EA’s). The data file contains household and women’s characteristics, as well as child health information for the most recent birth. For the present analysis, only those who had the most recent birth (within three years prior to the survey date) were considered. Permission to use the data for the purposes of the present study was granted by ICF international (U.S.) and Central Statistics Authority (Ethiopia) (http://dhsprogram.com/data/Access‐Instructions’). Ethical approval was also received by the University of Saskatchewan Behavioral Research Ethics Board.

### Measure of the outcome and exposure variables

For the regression analysis, four outcome variables were used. The first outcome was the child health service utilization score, which was constructed from the affirmative responses of six key child health interventions associated with the most recent birth: (1) ANC service (> 4 visits), (2) delivery of the last child at health facilities, (3) postnatal care services, (4) vitamin A intake, (5) iron supplementation and (6) intake of deworming by the index child. This outcome variable thus took a count form ranging from 0 to 6; taking a value of ‘0’ if the mothers’ response to the six indicators is “no,” and 6 if mothers respond ‘yes’ to all the six indicators. The three key health services (ANC, delivery, and postnatal care) were also used as separate outcome variables of their own to assess the likelihood of institutional delivery and postnatal care.

Health service utilization behavior is thought to depend on a set of individual, parental, household, and community-level characteristics. Thus, the exposure variables in the current analysis were categorized into three major groups: maternal and child factors (which includes, birth order, mothers’ education, age, work status, mother’s level of exposure to intimate partners violence, ever experienced pregnancy termination, parity, access to information/radio), household factors (which include non-monetary wealth index, religion, and type of family structure) and community variables (residence and type of region). The type of region was constructed based on clustering/grouping of the 11 regions based on their urbanization level and categorized as highly urbanized (Addis Ababa, Dire Dawa andHarari), medium-level urbanization (Tigray, Amhara, Oromia, SNNP, Gambella) and least urbanized (Afar, Benishangul Gumuz and Somali).

Most of the background variables (child’s sex, age, parental education, type of family structure, parity) were used the way they were coded in the original data. DHS constructed wealth index from selected key household assets and other characteristics that relate to economic status [25]. Intimate partner violence (IPV) was constructed from a set of dichotomous responses on a mother’s exposure to violence during a reference period of 12 months.

### Statistical analysis

The EDHS data are clustered (i.e., individuals are nested within households, and households are nested within the 645 enumeration areas/EAs) [25]. It is thus expected that mothers within the same cluster may have similarity. This violates the assumption of independence of observations across the clusters and, hence, limits the use of conventional regression as an outcome may be measured more than once on the same person [26]. Thus, a mixed effects regression was used. For the present analysis, the enumeration areas/EAs were used as clustering women respondents. mixed effects models are useful with data that have more than one source of random variability [26]. In this analysis, level one represents the individual (children characteristics), whereas level two is the cluster (community characteristics). Data were analyzed using STATA version 12 [27].

**Fig. 1:**
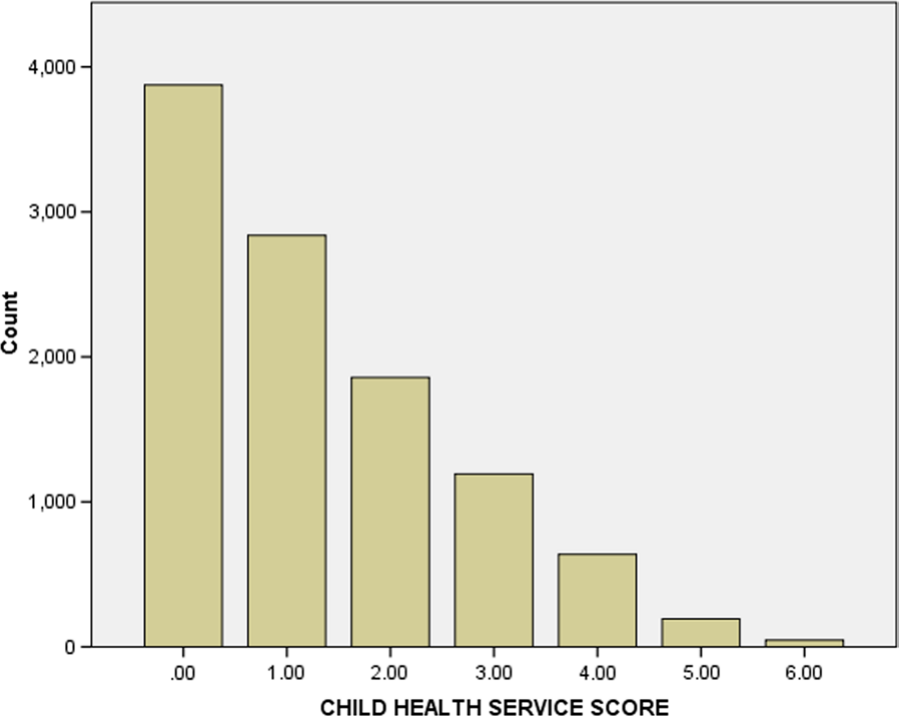
Distribution of the outcome variable: health service utilization scores, Ethiopia

Two sets of analyses were conducted. In the primary analysis, a mixed effect Poisson regression model was used to assess the determinants of service utilization score, which takes a form of count/rate, and skewed to the right (Fig. 1). In the secondary analysis, mixed effect logistic regression was used to assess the role of ANC in subsequent service utilization. The analysis began with checking if there was any multicollinearity between the explanatory variables using tolerance test/variance inflation factors (VIF). Using the routine Collin in Stata, a VIF > 10 or mean VIF > 6 represents severe multicollinearity [28]. Then, the bivariate association between child health service utilization and each potential predictor was examined. All predictors statistically associated with a *p* value of < 0.2 at bivariate level were subsequently included in the multivariable regression models. The model selection criterion was the Akaike Information Criterion (AIC), and the level of statistical error was set to be 5%. In the final model, we used a *p* value of < 0.05 to define statistical significance. The ratio of Deviance and Degree of Freedom (Deviance/DF) was used to test the model fitness [29]. The fitness of the model was also compared with a negative binomial regression model using AIC values and dispersion scores.

Further analysis of the continuum adherence to the health care service utilization was carried out using a mixed effect logistic regression model. The model hierarchically builds three separate models; model 1 contained predictors of ANC, model 2 adds ANC as a factor of the place of delivery, and model 3 included ANC and delivery place as key factors of postnatal care service utilization. All the analyses were weighted using the weight variable given by EDHS.

## Results

### Distribution of respondents by child health service utilization

Table 1 presents the results of chi-square analysis and percentage distribution of ANC, delivery, postnatal care service in Ethiopia by selected characteristics of the respondents and their under-5 children. The overall prevalence of ANC, delivery and postnatal care service utilizations were 34%, 26%, 12%, respectively. There were no significant sex differences in the three services (*p* value > 0.05). Health service utilization was higher among first-order birth compared to those in higher orders (*p* value = 0.000). The proportion of antenatal, delivery and postnatal care services was generally lower for older mothers, lower parity, and rural residents (*p* value < 0.001). Women living in small-sized households were better in utilizing the ANC (39.8%), institutional delivery (48.4%) and postnatal care services (22.3%). About 68.7%, 83.8% and 39.0% of women with secondary and higher education had at least four ANC visits, deliveries assisted by skilled professionals and postnatal care services, respectively (*p* value < 0.001). Nearly similar patterns were observed for the father’s education. It is noteworthy that the proportion of having had at least four ANC visits, institutional delivery and postnatal care steadily increased from the lowest wealth group (poorest/poorer) to the highest wealth group (richest/richer). More women from monogamous households (33.5%) made at least four ANC visits compared to women from polygamous households (22.9%). Similarly, more women from monogamous households had their most recent birth in health institutions (27.1%) and 11.7% of them had postnatal care. Larger proportions of women from Orthodox Christian attended the proper number of ANC visits, institutional deliveries and postnatal care services compared to those from other religions (*p* value < 0.001). There was a significant association between intimate partner violence (IPV) and the utilization of the three health services, where the proportion increased as the level of IPV increased.

**Table 1:** Results of bivariate relationship between the key child health service utilization and socioeconomic variables, EDHS 2016. Ethiopia

Variables*	At least 4 ANC visits Number (%)	*χ*^2^ (*p* value)	Institutional delivery	*χ*^2^ (*p* value)	Postnatal care service	*χ*^2^ (*p* value)
*Sex of the child*						
Male	1005 (31.9)	0.276	1237 (26.0)	0.232	566 (11.9)	0.249
Female	992 (33.7)		1190 (26.7)		509 (11.4)	
*Child’s birth order*						
First	480 (43.0)	0.000	841 (50.2)	0.000	284 (17.0)	0.010
Second and above	1517 (30.5)		1586 (21.0)		791 (10.5)	
*Age of the mother*						
15–24 y	425 (31.8)	0.000	679 (35.4)	0.000	251 (13.1)	0.000
25–34 y	1132 (36.3)		1273 (25.7)		588 (11.9)	
34+ y	440 (26.8)		475 (20.3)		237 (10.1)	
*Residence*						
Urban	512 (63.4)	0.000	803 (78.6)	0.000	380 (37.2)	0.000
Rural	1485 (28.1)		1623 (19.8)		695 (8.5)	
*Household size*						
0–3 member	330 (39.8)	0.000	439 (48.4)	0.000	202 (22.3)	0.000
4–6 members	1114 (35.2)		1324 (28.2)		571 (12.2)	
7 and above members	553 (26.3)		663 (18.4)		302 (8.4)	
*Education of mother*						
No education	975 (25.1)	0.000	990 (16.1)	0.000	487 (7.9)	0.000
Primary level	670 (39.5)		908 (37.3)		342 (14.1)	
Secondary & higher level	351 (68.7)		528 (83.8)		246 (39.0)	
*Education of father*						
No education	805 (25.9)	0.000	879 (18.6)	0.000	425 (9.0)	0.000
Primary level	754 (34.3)		857 (24.8)		353 (10.2)	
Secondary & higher level	438 (55.5)		690 (66.7)		298 (28.8)	
*Parity*						
0–3	1161 (39.1)	0.000	1589 (40.0)	0.000	664 (16.7)	0.000
4–6	562 (28.9)		579 (18.1)		282 (8.8)	
7 and above	274 (23.2)		259 (12.7)		129 (6.3)	
*Ever terminated pregnancy*						
No	1805 (32.5)	0.147	2208 (26.3)	0.193	967 (11.5)	0.090
Yes	192 (34.8)		218 (26.6)		108 (13.2)	
*Maternal autonomy*						
Yes, some autonomy	492 (35.8)	0.004	636 (30.8)	0.000	276 (13.4)	0.004
No autonomy	1505 (31.9)		1791 (25.0)		799 (11.2)	
*Wealth index*						
Poorest/poorer	606 (23.2)	0.000	648 (15.0)	0.000	241 (5.6)	0.000
Middle	373 (28.6)		438 (22.6)		194 (10.0)	
Richest/richer	1018 (46.6)		1341 (45.2)		640 (21.6)	
*Type of family structure*						
Monogamous	1710 (33.5)	0.000	2094 (27.1)	0.000	904 (11.7)	0.000
Polygamous	133 (22.9)		170 (17.0)		76 (7.6)	
*Intimate partners violence/IPV*						
No or low violence	410 (27.1)	0.000	436 (18.6)	0.000	166 (7.1)	0.000
Mild violence	607 (30.6)		706 (22.9)		335 (10.9)	
High violence	980 (37.7)		1284 (33.9)		574 (15.1)	
*Religion*						
Orthodox	938 (39.1)	0.000	1172 (36.8)	0.000	579 (18.2)	0.000
Muslim	585 (27.3)		734 (19.6)		276 (7.4)	
Others	474 (30.4)		521 (22.8)		221 (9.7)	
*Access to radio*						
Yes	777 (46.5)	0.000	946 (39.1)	0.000	483 (19.9)	0.000
No	1220 (27.6)		1481 (21.8)		593 (8.7)	
*ANC visits*						
< 4 times	–		840 (20.5)	0.000	447 (10.9)	0.000
> 4 times	–		1158 (58.0)		628 (31.4)	
*Place of delivery*						
Home	–		–	–	220 (3.2)	0.000
Institution	–		–	–	855 (35.2)	

Except for sex of the child, all the variables had a strong chi-square association with the respective outcomes (*p* < 0.001)

There was a strong significant association between ANC visits and subsequent delivery at health facilities. Among those who had > 4 times ANC visits, 58% of them delivered their last child at health facilities and only 20.3% for those who had < 4 times ANC visits (*p* value < 0.001). Also, 31.4% of those who had > 4times ANC visit received postnatal care services (*p* value < 0.001). Similarly, among those who delivered their last child at health facilities, 35.2% of them had postnatal care services compared to 3% for those who delivered at home (*p* value < 0.001).

As indicated in the method section, the distribution of the outcome variable followed a Poisson distribution where > 60% of the children had lower health service utilization scores (based on the median value) (see Fig. 1).

### Analysis of determinants of health service utilization score

Table 2 presents the results of mixed effect Poisson regression (bivariate and multivariable) for key explanatory variables of child health service utilization scores. It is noted that 13 of the 14 variables had significant bivariate associations with the outcome variable (*p* value < 0.05) and sex of the child had *p* value < 0.20.

**Table 2:** Results of bivariate (unadjusted) and multivariable (adjusted) mixed effect Poisson regression for explanatory variables of health service utilization score, Ethiopia

Variables	Unadjusted		Adjusted	
	RR (95% CI)	*p* values	RR (95% CI)	*p* values
*Random effect only model*			0.470 * (0.41–0.054)	< 0.05
*Fixed effects*				
Sex				
Male^RC^				
Female	0.97 (0.93–1.01)	0.061	0.98 (0.94–0.02)	0.399
*Birth order*				
First^RC^				
Second and above	0.62 (0.51–0.65)	0.000	0.42 (0.39–0.44)	0.000
*Age of the mother*				
15–24^RC^				
25–34	0.75 (0.71–0.78)	0.000	0.76 (0.72–0.81)	0.000
34+	0.85 (0.81–0.89)	0.000	0.43–0.39–0.47)	0.000
*Types of regions*				
Highly urbanized/more developed^RC^				
Medium-level urbanization	0.07 (0.04–0.11)	0.000	0.65 (0.56–0.76)	0.000
Low urbanized/least developed	0.01 (0.01–0.02)	0.000	0.31 (0.26–0.38)	0.000
*Education of mother*				
No education^RC^				
Primary level	1.44 (1.38–1.51)	0.000	1.49 (1.41–1.57)	0.000
Secondary and higher level	1.83 (1.72–1.95)	0.000	1.77 (1.64–1.90)	0.000
*Type of family structure*				
Monogamy^RC^				
Polygamy	0.89 (0.83–0.95)	0.001	0.87 (0.81–0.93)	0.000
*Parity*				
0–3^RC^				
4–6	0.57 (0.54–0.59)	0.000	0.10 (0.097–0.11)	0.000
7 and above	0.57 (0.54–0.61)	0.000	0.002 (0.002–0.003)	0.000
*Ever termination*				
No^RC^				
Yes	1.12 (1.05–1.19)	0.001	1.10 (1.02–1.17)	0.005
*Work status*				
No^RC^				
Yes	0.79 (0.76–0.83)	0.000	0.87 (0.83–0.91)	0.000
*Wealth index*				
Poorest/poorer^RC^				
Middle	1.23 (1.16–1.31)	0.000	1.12 (1.05–1.20)	0.001
Richest/richer	1.64 (1.54–1.74)	0.000	1.19 (1.14–1.27)	0.000
*Intimate Partners Violence/IPV*				
No or low violence^RC^				
Mild violence	1.11 (1.05–1.18)	0.000	1.06 (0.99–1.12)	0.071
High violence	1.24 (1.18–1.32)	0.000	1.10 (1.04–1.18)	0.001
*Access to radio*				
Yes^RC^				
No	0.74 (0.07–0.78)	0.000	0.86 (0.82–0.90)	0.000
*Constant*	0.07 (0.04–0.10)	0.000		
(Scale) offset	1			
*Deviance goodness of fit/DF = 1.31* *Number of groups: 642*				

*RC* Reference Category, *RR* rate ratio, *CI* confidence interval

In last two column of Table 2, the multivariable mixed effect Poisson regression results are presented. Goodness of fit for the model was checked using the ratio of Deviance and degree of freedom. The result shows that the model fits well (i.e., no substantial overdispersion). In such a model, if the ratio of Deviance/DF is closer to unity [1], the observed and fitted count values are matching, and hence, overdispersion is not a big concern. The output was also compared with a negative binomial regression model to see if the latter fits the data better. However, the estimated dispersion coefficient of the negative binomial regression coefficient (0.946; 95% CI 0.867–1.033) suggests that mixed effect Poisson regression is more appropriate. In addition, comparing the AIC of the two competing models suggests that the mixed effect Poisson regression has a slightly lower AIC (31,903.7) compared to the AIC of the negative binomial regression model (AIC = 32,475.8).

We started the analysis with only a random effect model to examine what service utilization score varied among mothers clustered together within the EAs before considering individual-level variations. The random effect only model had significant variance (0.470; CI 0.411–0.537). The covariate effect is interpreted as follows: For every one-unit increase in the covariate, the covariate has a multiplicative effect of ${e}^{\beta }$ (denoted as RR) on the expected mean of health care utilization score. The results indicate that, after controlling for other variables, the health service utilization score was lower among children of non-first birth order by 58% (${e}^{\beta }$=0.42, 95%CI 0.39–0.44) compared to first birth orders. The mean of service utilization score was lower by 24% and 57% for women in the age group 25–34 and 34+  (RR = 0.76, 95%CI 0.0.72–0.81 and RR = 0.43, 95% CI 0.39–0.47, respectively) compared to the younger women [15–24]. After controlling other variables in the model, the service utilization score was lower by 35% and 69% among those residing in medium and least urbanized regions, respectively (RR = 0.65, 95%CI 0.56–0.76 and RR = 0.31, 95% CI 0.26–0.38). Mothers with primary education had a higher mean score of health service utilization (RR = 1.185, 95%CI 1.125–1.248) compared to those with no education. Similarly, the service utilization score was higher for children with mothers of primary and secondary + education compared to those with no education. The rate of service utilization scores was lower by 13% for children from polygamous families (RR = 0.87, 95%CI 0.81–0.93) and by 13% for non-working women (RR = 0.87, 95%CI 0.83–0.91). The expected mean became lower by 14% for households with no access to radio (RR = 0.86, 95%CI 0.82–0.90). Similarly, mean significantly decreases with parities: lower by 90% for women having 4–6 parities (RR = 0.10, 95%CI 0.07–0.11) and by 98% for those with parities of 7+  (RR = 0.002, 95%CI 0.002–0.003).

The mean score of service utilization appeared to be higher by 1.21 times among mothers who had previous experience of terminated pregnancy (RR = 1.10, 95%CI 1.02–1.17) compared to the reference category. The mean of service utilization score became higher for those residing in more affluent households and those whose mothers experienced mild to high intimate partner violence.

### The role of ANC on subsequent delivery and postnatal care service utilization

In Table 3, results from three models of fixed-effect logistic regression analysis are given. Model 1 contains predictors of ANC visits; model 2 (place of delivery) contains all the variables in model 1 plus ANC; and model 3 (postnatal care service utilization) contains all the variables in the previous models plus place of delivery.

**Table 3:** Mixed effect logistic regression model for investigating the effects of ANC on subsequent use of health service utilization, Ethiopia

	Model 1 ANC	Model 2 Place of delivery	Model 3 Postnatal care
	AOR (95%CI)	AOR (95%CI)	AOR (95%CI)
*Random effect only model: EAs*	2.550 (2.135–3.044)	6.199 (5.257–7.310)	2.011 (1.673–2.416)
*Fixed effects*			
*Birth order*			
First^RC^			
Second and above	0.794 (0.663–0.951)	0.552 (0.447–0.683)	1.080 (0.886–1.315)
*Age of the mother*			
15–24^RC^			
25–34	1.363 (1.143–1.625)	0.972 (0.791–1.193)	1.197 (0.980–1.462)
34+	1.315 (1.036–1.670)	0.990 (0.749–1.309)	1.370 (1.042–1.802)
*Residence*			
Rural^RC^			
Urban	1.204 (0.863–1.680)	2.363 (1.589–3.513)	0.974 (0.713–1.330)
*Education of mother*			
No education^RC^			
Primary level	1.433 (1.233–1.666)	1.498 (1.267–1.772)	1.084 (0.901–1.303)
Secondary and higher	2.048 (1.61–2.603)	2.820 (2.088–3.809)	1.275 (0.989–1.644)
*Education of father*			
No education^RC^			
Primary	1.292 (1.122–1.489)	1.207 (1.028–1.417)	1.014 (0.852–1.207)
Secondary and higher	1.427 (1.176–1.733)	1.731 (1.378–2.176)	1.102 (0.892–1.360)
*Parity*			
0–3^RC^			
4–6	0.941 (0.798–1.110)	0.823 (0.680–0.995)	0.979 (0.801–1.196)
7 and above	0.809 (0.643–1.019)	0.910 (0.699–1.184)	0.837 (0.629–1.114)
*Ever termination of pregnancy*			
No^RC^			
Yes	1.161 (0.943–1.430)	1.187 (0.933–1.511)	1.160 (0.914–1.472)
*Autonomy*			
Yes^RC^			
No	1.030 (0.899–1.180)	0.926 (0.791–1.084)	1.017 (0.870–1.189)
*Wealth index*			
Poorest^RC^			
Middle	1.158 (0.963–1.393)	1.046 (0.854–1.281)	1.395 (1.101–1.769)
Richer	1.400 (1.155–1.697)	1.121 (0.905–1.388)	1.363 (1.064–1.745)
*IPV*			
High violence^RC^			
Mild violence	0.864 (0.749–0.996) _	0.957 (0.813–1.125)	1.096 (0.924–1.300)
No or low violence	0.997 (0.844–1.176)	0.899 (0.744–1.088)	0.968 (0.786–1.191)
*Access to radio*			
No^RC^			
Yes	1.374 (1.193–1.584)	1.043 (0.881–1.235)	1.609 (1.374–1.883)
*Mean wealth for community*	1.412 (1.249–1.596)	1.615 (1.401–1.862)	1.029 (0.906–1.170)
*Mean maternal education at the community*	1.025 (0.970–1.084)	1.125 (1.053–1.202)	1.008 (0.957–1.061)
*ANC service use*			
< 4 times^RC^			
> 4 times		2.892 (2.503–3.343)	1.490 (1.278–1.736)
*Place of delivery*			
Home^RC^			
Institutions			9.252 (7.691–11.128)
*Random effect coefficient*	0.839 (.664–1.062)	1.321 (1.052–1.659)	0.358 (0.238–0.542)
AIC Number of groups = 588	6724.66	5347.40	4811.56

*RC* Reference category

In the first model, seven factors have become significantly associated with ANC service utilization (*p* values < 0.05), namely birth order of the child, age of the mother, maternal education, paternal education, wealth index, access to radio and mean wealth at the community level (Table 3). Keeping other factors fixed, the odds of ANC decrease by 21% (AOR = 0.794; 95%CI 0.663–0.951) for children in second and above birth order compared to those in the first rank. The odds of ANC increase as the age of women increases. Women in the age group 25–34 and 34+ are 1.36 times (95%CI 1.143–1.625) and 1.32 times (95% CI 10.036–1.670) more likely to receive > times ANC, respectively, compared to young women aged 15–24. The odds of ANC visit is higher for mothers with primary (AOR = 1.433; 95%CI 1.233–1.666) and secondary + education (AOR = 2.048; 95%CI 1.61–2.603) compared to those who had no education. Similarly, the likelihood of ANC visits increases as the education of father increases.

Mothers living in richer and richest households had a significantly higher chance of having > 4 times ANC visits (AOR = 1.400; 95%CI 1.155–1.697) compared to those residing in poorer/poorest households. Those who had reasonable access to media (radio) had a higher likelihood of having > 4 times ANC visits (AOR = 1.374; 95%CI 1.193–1.584). It is also noted that the odds of having > 4 times ANC visits increase with increasing wealth index at the cluster level.

In model 2 (place of delivery), ANC was added to the model. Five individual and two community-level variables have become significantly associated with delivery service utilization (*p* < 0.05), namely birth order of the child, maternal education, paternal education, ANC visits, residence, mean wealth at the community level and mean maternal education at the cluster level. The effects of access to radio, wealth, and autonomy, which were significant in model 1, have become insignificant in this model (Table 3).

In model 2, the odds of institutional delivery decreased by 45% for children of higher birth order (AOR = 0.552; 95%CI 0.447–0.683) compared to first birth orders. Those residing in urban areas were 2.4 times more likely to deliver at health facilities (AOR = 2.363; 95%CI 1.589–3.513). The odds of ANC visits were higher for mothers with primary (AOR = 1.498; 95%CI 1.267–1.772) and secondary + education (AOR = 2.82; 95%CI 2.088–3.809) compared to those who had no education. Similarly, the likelihood of ANC visits increased as the education of father increases. The likelihood of delivering at health institutions increases with increased mean wealth and mean maternal education at the cluster level (AOR = 1.615 and AOR = 1.125, respectively). As expected, the ANC became the strongest factors of institutional delivery (AOR = 2.892; 95%CI 2.503–3.343).

In the last model (postnatal care), four variables were significantly associated with the outcome variable: birth order, education of mother, age of mother, wealth index, access to radio, ANC and place of delivery. Women’s likelihood of using postnatal care services was higher for subsequent births (AOR = 1.080; 95%CI 0.886–1.315) compared to their first birth. The likelihood of postnatal care service utilization is also higher by 1.37 times for older women (AOR = 1.37; 95% CI 1.042–1.802) compared to younger ones. Household wealth index appeared to be a strong factor associated with the propensity of postnatal care service utilization. The odds of postnatal care utilization were 1.40 times (AOR = 1.399; 95%CI 1.101–1.769) and 1.36 times (AOR = 1.363; 95%CI 1.064–1.745) higher for medium-level and richer/richest wealth households. The odds of postnatal care service utilization are higher for women who had access to radios (AOR = 1.609; 95% CI 1.374–1.883).

Model 3 further examined the combined effects of ANC and delivery service utilization on postnatal care needs of mothers. By holding the other factors fixed, both ANC and delivery service utilizations have become strongly associated with postnatal care service utilization. The odds of postnatal care service utilization were higher for those who had > 4 times ANC visits compared to those who had < 4 times ANC visits (AOR = 1.490; 95%CI 1.278–1.736). Similarly, the likelihood of postnatal care service utilization was higher for women who had institutional deliveries compared to those who had home deliveries (AOR = 9.252; 95%CI 7.691–11.128).

## Discussion

The study aimed at (1) assessing the key explanatory variables associated with the use of pre- and postnatal child care service scores for children under five years and (2) examining the role of ANC on the continuum adherence to institutional delivery and postnatal care service utilization in Ethiopia based on nationally representative data.

The finding indicates that the six components of health service utilization were very low by any standard, resulting in overall low utilization scores. For instance, it is noted that only a third of the most recent pregnancies had at least 4 ANC visits, only 26% of last births occurred in health facilities and only 12% received postnatal care services. A review of the national trends suggests a significant increase in ANC in Ethiopia with no corresponding increase in institutional deliveries. For instance, only 10% of births in EDHS 2011 survey was delivered by a skilled provider [30] compared to the 26% in 2016 EDHS survey. The reported rate was lower than the average for sub-Saharan Africa (42.9%) [31]. The implications of the low rates of these services on maternal and child health outcomes are important.

The result of the mixed effect regression analysis confirmed that health-seeking behavior for ANC and delivery care was significantly determined by the birth order of the index child. Though most studies in the subject have not focused on the influence of birth order on health-seeking behavior [32–34], the few available pieces of evidence generally indicate that mothers tend to invest less in health and child well-being for higher-order births than first-order births [32, 35, 36]. In most Ethiopian cultures, first birth is the most wanted, often accompanied by colorful ceremonies than any subsequent births. The finding implies the need for continued education on the importance of adequate prenatal and postnatal care to higher-order births.

The age of women is another demographic variable predicting the rate of child health service utilization score. The results of mixed effect Poisson regression analysis showed that older mothers have lower odds of adhering to pre- and postnatal health care service use. In relation to this, women of higher parity were less likely to have higher service utilization scores. Age of women and parity are the most commonly reported factors impacting both ANC and delivery care service utilization in Ethiopia and developing countries context [37–39]. One plausible reason for the inverse relationship between parity (and age of women) and service utilization could be increased limitations of resources and time as the number of children to be raised increases. Some studies also attribute this to increased confidence of mothers as they have more children [13, 40].

The positive effect of maternal education on a mother’s chance of utilizing child health services is strong. A comparable national report showed that 69.6% of births to women with a secondary school education occurred in a health facility and with skilled assistance compared to 4.3 percent of births for women with no education[30]. Nigussie et al. [12] found that women with a higher level of education (secondary and above) were 10.6 times more likely to use safe delivery services than those with lower education levels. Another study in North Gondar Zone, Ethiopia, reported that the use of skilled birth attendants was significantly influenced by the level of education. Other studies also made similar conclusions [9, 10, 41]. One of the reasons for this might be education promotes certain dimensions of autonomy such as freedom of movement, decision making power and control over finance can exert a strong influence over service use and service choice [42]. Education generally creates favorable self-selection bias where knowledgeable or better-educated women invest in health services since they possess superior knowledge about the associated benefits to their personal health, as well as the health of their children [32].

Our study disproves the general notion that paternal education has little or no effect on child health care [43], which has led many researchers to mainly focus and, at times, exclusively report on the importance of maternal education in studying health inequities [43, 44]. Results from the mixed effect logistic regression model showed that women living with educated fathers are more likely to use ANC and delivery services. This is not surprising as some previous studies have reported that educated fathers are more likely to involve in child well-being such as diet/nutrition, exercise, play, and parenting behaviors, which contribute to the overall health and well-being of their young children [45, 46]. Additionally, educated fathers provide a higher household income, more freedom and supports, higher social status and stability, and more opportunities for their families (wives and children) to access health care services [42, 47, 48].

It is noted that women with poor access to media had lower chance of utilizing child health services. An Ethiopian study conducted by Mehari[49] showed that women who frequently watch TV were more likely to receive skilled assistance during delivery. A study in India concluded that women’s exposure to information through radio, television and newspaper significantly increased the utilization rates of skilled delivery services[50]. As most Ethiopian women have no education, they benefit little from printed media, getting information through an informal way or radio. Further, the proportion of Ethiopian women having radios in their households is low (about 33%) [15].

Significant disparities are noticed in the likelihood of utilizing the health services across household wealth quintiles. Women in the wealthiest households are more likely than women in the poorest households to have used child-related health services. The Central Statistics Authority’s report [30] showed that 45% of women in the highest wealth quintile used assistance from SBAs compared with 2% percent of women in the lowest wealth quintile. Another study in Ethiopia reported that women in the rich and richest wealth group were 1.8 and 3.4 times higher, respectively, to use skilled assistance [11]. Similarly, Mehari [49] also reported a strong association between wealth index and the utilization of skilled delivery care services.

We found significant association between service utilization score and type of regions (i.e., base on clustering the 11 regions into three groups based on their level of urbanization). The finding is consistent with previous studies conducted in Ethiopia and elsewhere. It was found that mothers in less urbanized regions are less likely to received ANC during pregnancy, and subsequently, they will have a lower likelihood to adhere to the delivery and postnatal care services. A study based on national-level data from 29 sub-Saharan African countries [9], for instance, reported the odds of urban women delivering in a health facility more than doubled the odds of rural women delivering in a health facility. According to EDHS 2011, 49.8% of those urban residences deliver at health centers, while only 4.1% of the rural residents deliver at health facilities. Bell and Colleagues [39] observed similar trends for Bolivia, Malawi, Indonesia, Bangladesh and the Philippines in their analysis of data from their Demographic health surveys.

Further analysis of the three key health services (ANC, delivery, and postnatal care) indicates consistent positive impacts of ANC on the continuum of utilizing delivery and postnatal care services. Interestingly, of all the potential predictors entered in the second regression model, ANC had the greatest effects on both institutional delivery and postnatal care service utilization. This means that if women get access to ANC services during her pregnancy, she is more likely to give birth at the health institution compared to those who did not have the prescribed number (> 4) of antenatal visit. This adherence was further examined in the third regression model where both ANC and institutional delivery were combined to contribute the greatest effect on the likelihood of mothers’ utilization of postnatal care services in the first two months after delivery. Controlling for all other variables in the model, the odds of postnatal care service utilization were 1.49 times and 9.25 times greater for those who attended ANC and delivered their most recent baby in health institutions. The findings are consistent with a recent study conducted based on data from 58 Demographic and Health Surveys from 29 sub-Saharan African countries, which confirm that ANC attendance was predictive of facility-based delivery [9].

Finally, the present study has both limitations and strengths. Regarding the limitations, the cross-sectional nature of the EDHS survey limits the ability to draw a cause-effect relationship between the exposure variables and the outcomes. Because most of the survey respondents (mothers) had no education, there might be some response bias and measurement errors during data collection. On the other hand, the most plausible strength of the present study is that the findings can be generalized to the entire population/regions of the country. Thus, its use in monitoring and evaluation of health services and related programs is high. Since comprehensive outcome measure and more sophisticated statistical analysis are used, the findings could benefit program implementation at community level.

## Conclusion

The findings of the present study implicated that maternal and child health services appear as continuum actions/behavior where utilization of one affects the likelihood of the next service types. The study indicated that promoting proper antenatal care services is very beneficial in increasing the likelihood of mothers utilizing subsequent services such as delivery and postnatal care services. As Ethiopia is striving to achieve the SDG targets to reduce the current unacceptably high maternal and child mortality, more efforts should be made to improve women’s access to education with more attention to younger women and those living in polygamous marriage, increasing access to information, and improving household economic status. Increasing women’s empowerment and participation in their households and communities might enable them to achieve control over their own and child’s health. Narrowing down the regional disparities in terms of their level of urbanization would help increase households’ access to a wide range of resources.

## Data Availability

The datasets used for this study are made available from ICF international upon request. Please visit: https://dhsprogram.com/data/new-user-registration.cfm.

## Abbreviations

ANC: Antenatal Care
CSA: Central Statistics Authority
DHS: Demographic and Health Surveys (DHS).
EDHS: Ethiopian Demographic and Health Surveys
ENA: Essential Nutrition Action
GDP: Gross Domestic Product
IPV: Intimate Partners Violence
RR: Relative Risk
SDGs: Sustainable Development Goals
SES: Socioeconomic Status
US: United States
WHO: World Health Organization
